# Medfly *Ceratitis capitata* as Potential Vector for Fire Blight Pathogen *Erwinia amylovora*: Survival and Transmission

**DOI:** 10.1371/journal.pone.0127560

**Published:** 2015-05-15

**Authors:** Mónica Ordax, Jaime E. Piquer-Salcedo, Ricardo D. Santander, Beatriz Sabater-Muñoz, Elena G. Biosca, María M. López, Ester Marco-Noales

**Affiliations:** 1 Instituto Valenciano de Investigaciones Agrarias (IVIA), Centro de Protección Vegetal, Ctra. Moncada—Náquera, km 4.5, 46113, Moncada, Valencia, Spain; 2 Universitat de València, Departamento de Microbiología y Ecología, Av. Dr. Moliner, 50, 46100, Burjasot, Valencia, Spain; Ghent University, BELGIUM

## Abstract

Monitoring the ability of bacterial plant pathogens to survive in insects is required for elucidating unknown aspects of their epidemiology and for designing appropriate control strategies. *Erwinia amylovora* is a plant pathogenic bacterium that causes fire blight, a devastating disease in apple and pear commercial orchards. Studies on fire blight spread by insects have mainly focused on pollinating agents, such as honeybees. However, the Mediterranean fruit fly (medfly) *Ceratitis capitata* (Diptera: Tephritidae), one of the most damaging fruit pests worldwide, is also common in pome fruit orchards. The main objective of the study was to investigate whether *E*. *amylovora* can survive and be transmitted by the medfly. Our experimental results show: i) *E*. *amylovora* can survive for at least 8 days inside the digestive tract of the medfly and until 28 days on its external surface, and ii) medflies are able to transmit the bacteria from inoculated apples to both detached shoots and pear plants, being the pathogen recovered from lesions in both cases. This is the first report on *E*. *amylovora* internalization and survival in/on *C*. *capitata*, as well as the experimental transmission of the fire blight pathogen by this insect. Our results suggest that medfly can act as a potential vector for *E*. *amylovora*, and expand our knowledge on the possible role of these and other insects in its life cycle.

## Introduction

Phytopathogenic bacteria cause annually very important losses in major crops and fruit trees, producing serious economical damage. Epidemiological studies have been mostly focused on plant-pathogen interactions, excluding the role of other organisms in disease dissemination. Insects are often neglected as ecological players, but many plant diseases become more severe and detrimental in the presence of specific or nonspecific insect vectors that spread the pathogen to new hosts [1]. Nowadays, there is a trend to investigate plant diseases at a community level [2], looking for a better understanding of the interactions and associations of the bacteria with other organisms in the environment.

The bacterium *Erwinia amylovora* is the causal agent of fire blight, a destructive and highly infectious disease of apple, pear and other rosaceous plants. The name of the disease is derived from the characteristic dark discoloration of affected plant tissues, as if they were burnt. Fire blight causes dramatic losses worldwide, and remains as a disease difficult to control due to the lack of fully efficient chemical and biological treatments and the ability of *E*. *amylovora* to persist in nature and to spread in diverse ways [3]. *E*. *amylovora* cells are usually disseminated by insects, rain, wind or wind-driven rain (as aerosols) to open blossoms, and also to shoots, tender leaves and fruits [3, 4]. In a recent review on fire blight by Billing [5], the author concludes that some aspects of *E*. *amylovora* life cycle and fire blight epidemiology rest on uncertainty. Although it is considered that *E*. *amylovora* uses blossoms as the main route of infection, natural openings or wounds can also provide entryways into the plants [6]. Further, there are many insects presumably associated with the spread of fire blight [7, 8, 9, 10], although their exact role and the *E*. *amylovora* survival in/on these insects is poorly understood, specially in non-pollinating agents [4, 11].

There is a wide presence of non-pollinating insects in pome fruit orchards throughout summer, late spring and early autumn [12]. An important group of these insects are the fruit flies (family *Tephritidae*), which have a particular relevance in agriculture [13]. The Mediterranean fruit fly, or medfly, *Ceratitis capitata* (Wiedemann) is one of the most destructive fruit pests worldwide and is considered the most important invasive species throughout the world [14, 15, 16, 17]. The medfly has been extremely successful at invading and settling new areas, particularly due to its polyphagous diet, causing damage in nearly 400 plant species, and its liberal host acceptance behavior, rapid population growth, and high tolerance for a wide range of climates [15]. In addition, the medfly can be spread via the local sale or exportation of fruit, and it can fly at least 20 km, which greatly complicates the efforts to control this insect [18, 19]. Thus, the medfly is now spread throughout more than 70 countries, and it is considered a quarantine pest in many of them [16]. However, up to date scarce reports on fruit flies as vectors of plant and human diseases are available [20, 21, 22]. Since high densities of *C*. *capitata* are usually found in apple orchards [12], our main objective was to investigate whether *E*. *amylovora* can survive and be transmitted by *C*. *capitata*. Because the predilection of medfly for apple fruit as the most suitable host [23], we selected these fruits, which could act as a vehicle for *E*. *amylovora* dissemination [24, 25, 26]. In the light of results obtained, we consider that medfly can not be neglected as a potential vector for *E*. *amylovora*, since this pathogen can survive in/on *C*. *capitata* and be transmitted to plant material causing disease symptoms.

## Materials and Methods

### Bacterial strains and growth conditions

Two reference strains of *E*. *amylovora*, CFBP1430 (from *Crataegus oxyacantha*, France) and NCPPB2080 (from *Pyrus communis*, New Zealand), and their respective green fluorescent protein (GFP) and red fluorescent protein (DsRed)-labeled transformants were used. Green fluorescent transformants 1430-GFP1 and 2080-GFP3 were previously obtained [24]. Transformants 1430-DsRed2 and 2080-DsRed1 were obtained in this study by transformation of competent cells with plasmid pDs-Red, which confers ampicillin resistance and red fluorescence. The colony morphology of DsRed-tagged strains on growth media was the same of the parental strains but with a pink-red colour provided by the pDs-Red plasmid. There were not differences in pathogenicity and severity of symptoms when DsRed-transformants were inoculated in immature pears cv. Devoe. Green-fluorescent transformants were used to monitor the bacteria on external surfaces of flies, while red-fluorescent ones were used to monitor them inside the insects, since in the preliminary experiments it was observed that fly tissues show a green fluorescence interfering with that of GFP-bacteria.

The semi-selective media CCT [27] and RESC (Recovery-*Erwinia amylovora*-Stressed-Cells) [28], and the general media King’s B (KB) [29], Luria-Bertani (LB) and sucrose nutrient agar (SNA) [30], all of them in solid and liquid formulation, were used for growth of *E*. *amylovora*. In the case of GFP or DsRed-tagged strains, media were supplemented with tetracycline (12.5 μg/ml) [24] or ampicillin (100 μg/ml), respectively. Incubation conditions were 26°C for 48h, at 50 rpm of shaking in the case of liquid media. For the isolation and/or recovery of challenged *E*. *amylovora* cells, incubation period was extended up to 7 days.

### *C*. *capitata* strain and rearing conditions

Medfly pupae were obtained from the Entomology Laboratory of IVIA, from colony IVIA2002 [31]. Adults were maintained in a 20x20x20 (cm) Perspex cage at 25 ± 40°C, 75 ± 5% RH and a 16:8 h (L:D) photoperiod in an environmental chamber (MLR-350, Sanyo). Standard food consisted of a mixture of sugar (from sugar beet, *Beta vulgaris* L.; Azucarera Ebro, SL, Madrid, Spain), hydrolyzed yeast (Biokar Diagnostics Co., Pantin, France) (4:1; wt:wt) and water. Sexually mature medflies (5–7 days old) were used for all assays.

### Plant material

Royal Gala apple fruits from organic culture were used in acquisition and transmission experiments (see below). They were disinfected with a 60% sodium hypochlorite solution for 5 min, washed 3 times for 15 min each with sterile distilled water, and then dried [24]. Detached young pear shoots of cv. Conference were disinfected with 50% ethanol for a few seconds, followed by 3 washings of 10 min each with sterile distilled water, left to dry and their bases introduced into sterile 1.5% agar [24]. Whole pear seedlings used for transmission experiments were obtained from Conference pear seeds. Following the protocol of Santander et al. [32], the seeds were disinfected with sodium hypochlorite 3% (wt:vol) for 5 min, washed with sterile water, dried, and stratified in wet river sand at 4°C. After 1–3 months, the seeds were transferred to an autoclaved nutritive substrate (black and white peat, sand and perlite) and incubated in an environmental chamber (MLR-350, Sanyo) for two months (stem length 8–16 cm). Immature apple fruits of cv. Golden Delicious and loquats cv. Argelino (2–4 cm diameter) were disinfected with a 30% sodium hypochloride for 1 min, washed 3 times for 10 min each, and then dried [24, 33,] before inoculation.

### Acquisition of *E*. *amylovora* by the medfly from inoculated mature apples

Two disinfected mature apples were challenged in cages (20x15x10 cm) in which they were placed in opposite positions (peduncle or calyx face up). Several wounds were made in each apple: six cuts of 1.5 cm in the central area of the fruit and five cuts in the area surrounding the peduncle or calyx. Each cut was inoculated with 20 μl of an *E*. *amylovora* suspension at 10^7^–10^8^ CFU/ml in PBS buffer. Afterwards, medflies were introduced into the cages: 25 males and 25 females, 5 males and 5 females, 2 males and 3 females, or only one male or one female, depending on the assay. For 48 h, all cages were maintained under conditions favorable for both *E*. *amylovora* and *C*. *capitata* (26 ± 2°C, 12-hour light/dark cycle, 75–85% RH) in an environmental growth chamber (MLR-351, Sanyo). Acquisition experiments were performed in duplicate and repeated independently for each *E*. *amylovora* strain and batch of medflies. After the period for acquisition of the bacterium by the medfly, a) the inoculated apples were removed from half of the cages containing 50 medflies for being used in survival studies (see the next section), and b) in the remaining cages, the medflies were captured and transferred to other cages with healthy plant material for transmission assays (see further below for the two sections on transmission).

To verify acquisition of the pathogen, some insects were analyzed for the presence of *E*. *amylovora* culturable cells. Groups of 3–5 medfly individuals were crushed in 2.5 ml TNES buffer [31], and these extracts and their dilutions were plated onto solid CCT medium or enriched with 1 ml CCT broth. In addition, 300 μl aliquots of each medfly extract were subjected to an insect DNA extraction protocol [31] before performing a specific PCR analysis to detect *E*. *amylovora* [34].

### Survival of *E*. *amylovora* on/in medflies and cages

Immediately after the acquisition stage and after removing the inoculated apples from 16 cages with 50 medflies per cage, we began to monitor the survival of the bacterium on the medfly over 28 days. The survival of *E*. *amylovora* populations was monitored at 7, 14, 21 and 28 days after contact with the inoculated *C*. *capitata*. Throughout the challenge period, the medflies were fed with 10% sterile sucrose, as lifespan of starved flies is of 1–2 days only [35]. Weekly, living medflies (approximately 10–15 insects) were analyzed for the presence of *E*. *amylovora* by the cultural and PCR methods described above, plating up to 1 ml of medfly extract to improve the detection limit (<1 CFU/fly). *E*. *amylovora*-like colonies were identified by PCR, and the pathogenicity of representative colonies was verified by inoculation into immature apples, loquats, and pear shoots [36]. If the culturability analysis was negative but PCR was positive, the corresponding medfly extracts were subjected to recovery assays (see further below). The survival experiments were performed in duplicate and repeated independently for each *E*. *amylovora* strain.

Food and drinking water from cages containing flies were analyzed 8 days after the acquisition period to discard their possible contamination with *E*. *amylovora* and, therefore, the continuos acquisition of the bacterium by the medflies over time. For this purpose, samples of food, drinking water, and also aborted eggs, regurgitated food, and from the walls of the cages, were taken with sterile swaps, which were immersed in liquid CCT medium (supplemented with antibiotics when required) and incubated at 26°C.

### Integrated recovery protocol for non-culturable *E*. *amylovora* cells

When no *E*. *amylovora* colony was found on solid medium after plating 1 ml of medfly extract, recovery assays were carried out *in vitro* and *in vivo* in accordance with previous works [24, 33, 37]. Thus, an enrichment of the extracts was performed by adding KB and CCT broth (1:1), followed by plating onto solid CCT medium, for the *in vitro* assays. *In vivo* recovery was based on the inoculation of susceptible plant material, either immature apples or loquat fruits, or detached pear shoots. A volume of 15 μl of medfly extract was inoculated per cut (four cuts per immature fruit and one cut per young leaf) and the plant material was regularly examined throughout 15 days. A suspension of the strain CFBP1430 at 10^8^ CFU/ml in PBS buffer was used as a positive control and PBS and TNES buffers as negative ones.

### Transmission assays of *E*. *amylovora* through the medfly

#### (i) Transmission to mature apples

Medflies that had the opportunity to acquire the bacterium from inoculated apples were transferred to other cages with two healthy mature apples in opposite orientations. These apples had been injured by performing wounds (1–1.5 cm), distributed as described above, to mimic fruits in orchards where they are subjected to injuries from birds, insects, worms, hail, rain, wind, and other biotic or abiotic factors. Throughout the five-day transmission period, the cages were maintained under the same conditions as those used for the acquisition period. Transmission assays were performed in duplicate and repeated independently for each *E*. *amylovora* strain. Once the transmission period had elapsed, live medflies, eggs, and apples were analyzed to detect *E*. *amylovora*. Insects were processed as bulk samples as described above, in groups of one to five. Eggs were frozen, crushed in 300 μl TNES buffer, and analyzed by PCR after DNA extraction as described for the medflies.

Apple fruits were analyzed individually. First, each fruit was washed in 10 ml PBS buffer to detect external *E*. *amylovora* cells. Second, flesh layers (approximately 0.5 cm beneath each wound) were removed, crushed in 2.5 ml AMB buffer [38] and processed for detection of internal *E*. *amylovora* cells. Washings and flesh extracts from each apple were analyzed separately by plating directly onto CCT medium or onto KB after enrichment in KB or CCT broth in proportion 1:1 and by specific PCR amplification [34] after DNA extraction [39]. If *E*. *amylovora*-like colonies were observed, they were PCR identified and their pathogenicity verified [36] as indicated above. The remaining washings and extracts were frozen at—20°C in 30% (vol:vol) glycerol.

#### (ii) Transmission to pear shoots and plants

Batches of approximately 50 medflies that had the opportunity to become contaminated with *E*. *amylovora* from inoculated apples were transferred to cages containing detached pear shoots or whole plants of cv. Conference. These assays were performed with either intact leaves or ones that had been cut to the main vein following an inoculation standard procedure [36]. Incubation conditions were as in the acquisition stage. The transmission period ranged between 5 and 14 days, depending upon the evolution of symptoms. After the incubation period, the live medflies were analyzed by cultural techniques and by PCR as previously described. Plant material (shoots or whole plants) was processed individually. The symptomatic parts of each leaf were processed by comminuting them in 1–1.5 ml AMB buffer [36], and after few minutes, the extracts were plated onto solid media, enriched with broth or analyzed by PCR as for mature apples. *E*. *amylovora*-like colonies were also confirmed by PCR.

### Monitoring of *E*. *amylovora* on/in medflies by fluorescence microscopy

Over 20 live medflies from the survival and transmission assays that were challenged with the fluorescent transformants of *E*. *amylovora* strains were visualized using a Nikon ECLIPSE E800 epifluorescence microscope with filters B-2A (EX 465–495 nm, DM 505 nm, BA 515–555 nm) and/or G-2A (EX 450–490 nm, DM 505 nm, BA 520 nm). To detect *E*. *amylovora* cells on medfly surfaces, intact individuals were placed on hollow slides and examined under epifluorescence microscope. The same flies were also observed after being slightly crushed with cover slips. To evaluate the possible internal location of *E*. *amylovora* in medflies through the time, groups of three medfly individuals challenged with DsRed-tagged *E*. *amylovora* cells (since native fluorescence of fly tissues interferes with GFP fluorescence) were analyzed at 4, 8 and 24 h throughout the acquisition period, and also 1, 4, 8 and 15 days after that period. Medflies were processed and sectioned with a cryostat Leica CM1510 S (Leica Biosystems) as follows. Wings and legs were removed, heads detached from the body and both of them immersed by separate into a sterile 30% (wt/vol) sucrose solution in 10 mM PBS pH7.2, at 4°C for 48-72h. Previously to cut abdomen and thorax sections with the cryostat, medfly bodies were embedded in Tissue-Tek^®^ O.C.T.^TM^ Compound (Sakura Finetek) and frozen to -30°C. Tissue sections were observed with the epifluorescence microscope.

An enrichment in CCT broth supplemented with ampicillin was performed in those tissue samples that were negative for red-fluorescent bacterial cells by microscopic observation. Thus, slides with these samples were placed into sterile Petri dishes, covered with the semi-selective medium and incubated under favorable conditions for 16 h before plating the enriched liquid on KB.

### Statistical analysis

*E*. *amylovora* cell counts (after log-transformation) were analyzed as the means of two replicate samples from at least two independent experiments (n≥4). Significant differences were determined by analysis of variance (ANOVA). Fixed main factors considered in the survival studies were the experiment, strain and duration of insect contact. For the apple transmission assays, the factors were the experiment, strain, part of the apple fruit, medfly sex and location (inside/outside) in/on the fruit. For the shoot and plant transmission assays the factors were the strain, shoot or plant and leaf necrosis. Two assumptions to apply this test were checked. First, the sample should meet the assumption of normality of the quantitative response variable (CFU), so it was normalized transforming it logarithmically. Second, the assumption of homogeneity of variances was verified. Data below the detection limit of the plate counts were not included in the analysis. Differences were considered significant for p values <0.05.

## Results

### *E*. *amylovora* can be acquired by and survive on/in medflies

The medflies were observed walking near the wounds made on mature apples inoculated with *E*. *amylovora* and feeding on the inoculum drops, as expected by its feeding behavior and attraction by odor clues [14, 15, 35, 40]. These contaminated apples did not show any fire blight symptom throughout the challenge period, despite the fact that *E*. *amylovora* colonies were always re-isolated from the apples at levels similar to the inoculum doses. Analyses of the medflies after 48 h of contact with the apples showed positive results for *E*. *amylovora* detection by cultural (in all media assayed) and specific PCR methods, with pathogen concentrations ranging from 10^4^ to 10^6^ CFU/medfly.

**Fig 1:**
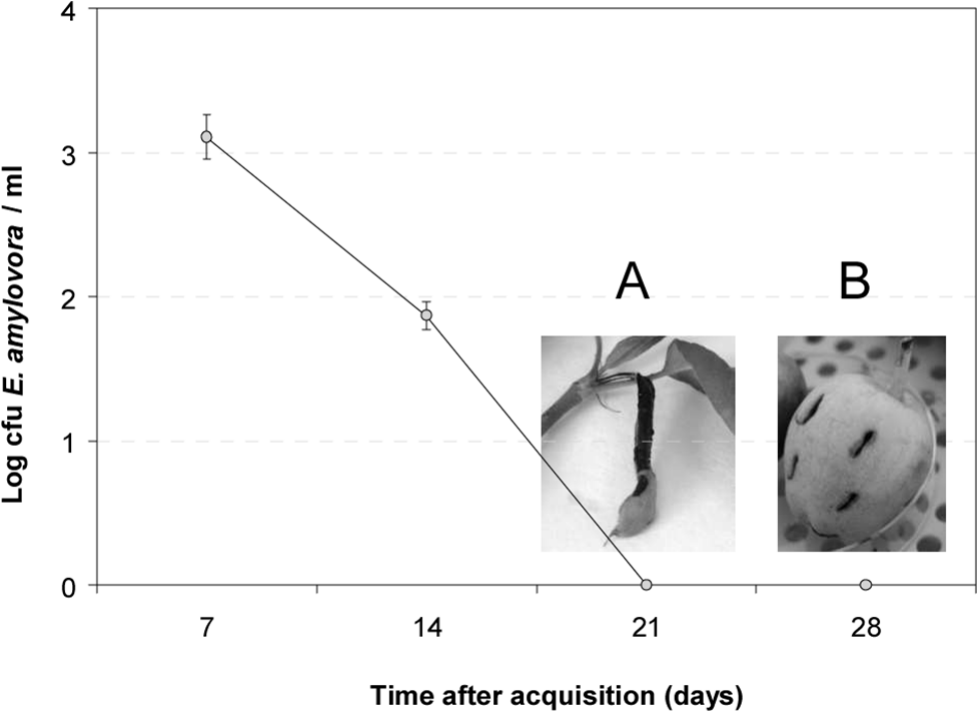
Survival of *E*. *amylovora* strain CFBP1430 on *C*. *capitata*. Culturable cells of *E*. *amylovora* were recovered up to 14 days after contact with medflies (detection limit < 1 CFU/medfly). However, *C*. *capitata* extracts containing non-culturable *E*. *amylovora* cells obtained after 21 or 28 days produced symptoms in detached pear shoot (A) (picture taken at 7 days post inoculation, dpi, showing necrosis) and immature apple (B) (picture taken at 14 dpi, showing necrosis and sinking of wound edges). Bars corresponding to SD are less than 0.19.

After 7 and 14 days of pathogen being in contact with the medflies, *E*. *amylovora*-like colonies were recovered from medflies on CCT solid medium and then identified by PCR (Fig 1). There were no significant differences among the assayed strains (p>0.05) (S1 Fig). Colonies of *E*. *amylovora* were easily distinguishable on CCT and RESC media from native bacteria of the medflies, such as *Serratia marcescens* and *Providencia rettgeri*, identified by partial 16S ribosomal DNA sequencing. Inoculation of *E*. *amylovora* cells re-isolated from medflies into immature apples and loquats showed that the bacterium maintained its pathogenic potential after contact with the insect (data not shown).

However, no *E*. *amylovora*-like colonies were observed in medfly extracts after 21 and 28 days of contact between the bacteria and the medflies (detection limit <1 CFU/medfly) (Fig 1) despite PCR results were positive. To determine whether *E*. *amylovora* had died or lost the culturability, these medfly extracts were subjected to *in vitro* and *in vivo E*. *amylovora* recovery assays. *In vitro* assays based on initial enrichments in KB and CCT broth were unsuccessful in recovering culturability of nonculturable *E*. *amylovora* cells, even after incubation periods longer than one week. In contrast, recovery was achieved by extract passage through susceptible plant material such as immature apple and loquat fruits or detached young pear shoots. After inoculation with these medfly extracts, both shoots and fruits showed fire blight symptoms at 7–14 days post-inoculation (dpi) (Fig 1A and 1B). Approximately 10^3^ *E*. *amylovora*-like CFU/lesion were recovered from small necrotic areas and 10^7^ CFU/lesion from extensive necroses, regardless of the type of plant material or extract inoculated. Presumptive *E*. *amylovora* colonies were confirmed by PCR. In all cases, positive controls showed severe fire blight symptoms at 5–7 dpi, and negative controls showed no signs of disease.

Interestingly, *E*. *amylovora* was not detected, after direct isolation or after enrichment in CCT, neither in food, water for drinking, aborted eggs or regurgitated food, nor in the walls of the cages used for challenges.

### The medfly transmits *E*. *amylovora*

#### (i) Transmission to mature apples

Medflies with *E*. *amylovora* cells acquired from inoculated apples were enclosed with healthy apples for 5 days. The numbers of *E*. *amylovora* cells carried per medfly after that period ranged from 10^3^ to 10^5^ CFU, and the plant pathogen was never detected in the medfly eggs. Challenged apples did not develop fire blight symptoms either on the peel or in the flesh during the assayed period; however, cultural and PCR analysis of these apples after 5 days revealed that *E*. *amylovora* had been transferred to the fruits. External and internal *E*. *amylovora* populations transmitted to the mature apples were quantified as approximately 10^5^–10^4^ and 10^4^–10^3^ CFU/fruit, respectively. Furthermore, *E*. *amylovora* cells recovered from the recipient apples were pathogenic when inoculated into susceptible plant material, which developed typical fire blight symptoms.

**Fig 2:**
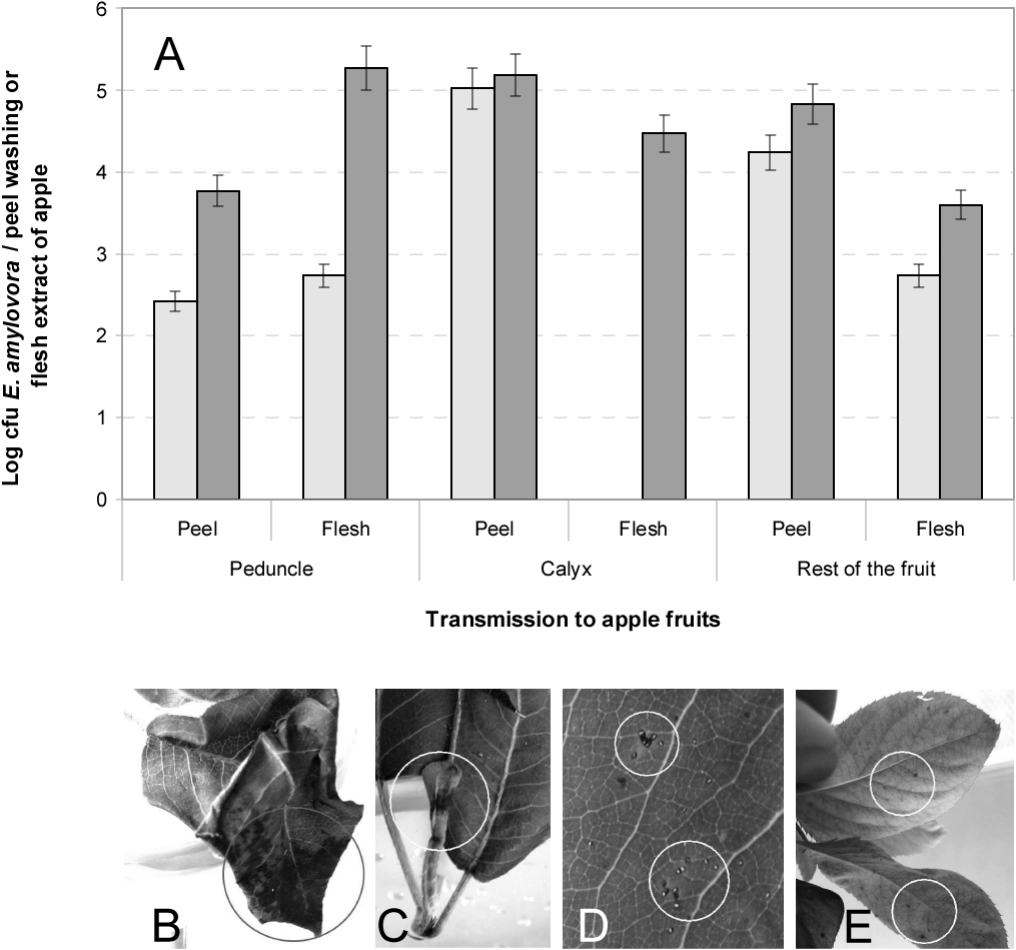
Transmission of *E*. *amylovora* to mature apples by *C*. *capitata*. Number of CFBP1430 strain CFUs counted after processing washings of fruits (one piece/10 ml PBS) or flesh extracts (obtained by crushing flesh layers in 2.5 ml AMB buffer) after transmission by both males (light grey bars) or females (dark grey bars) (SE is represented by vertical lines) (A). Transmission to detached young pear shoots, showing necrotic lesions in intact (not pre-injured) leaves (B, C). Medfly eggs embedded in the leaf tissues (D). Transmission to potted pear plants showing black spots on intact leaves (E).

**Fig 3:**
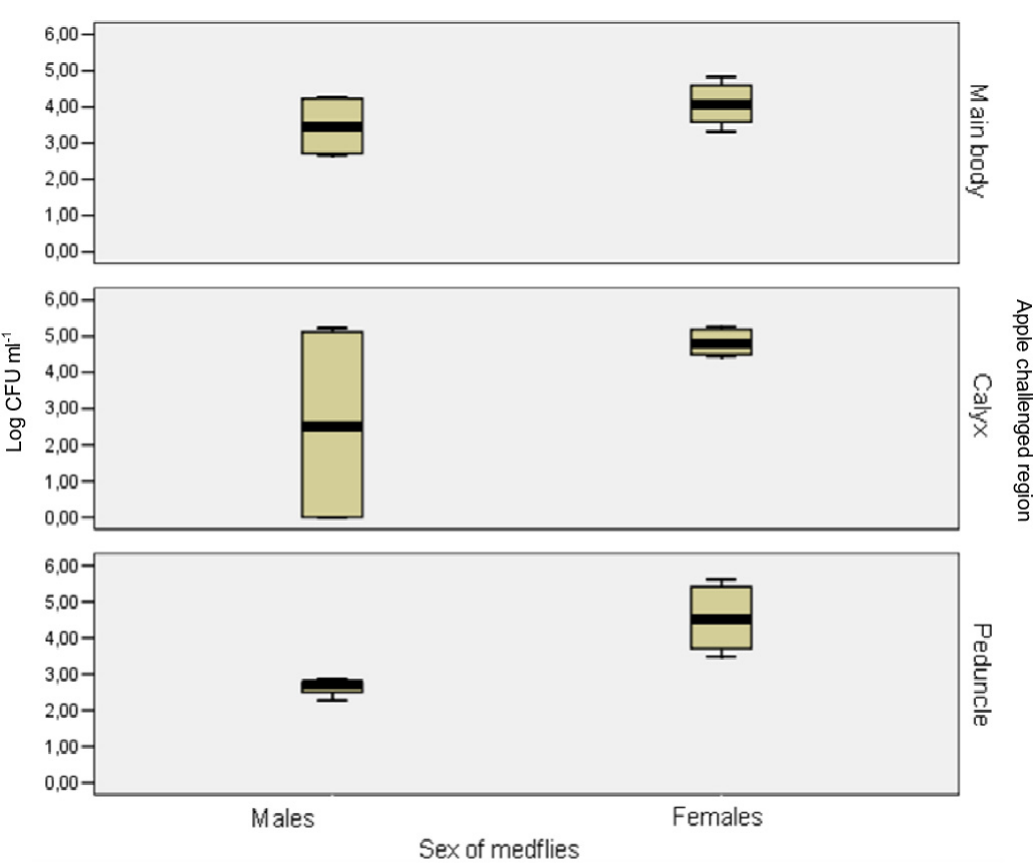
Boxplot of CFU mL^-1^ of *E*. *amylovora* from calyx, peduncle or the rest of the fruit of mature apples after transmission by *C*. *capitata*. Colonies of CFBP1430 strain were counted from mature apples after transmission experiments by males or females *C*. *capitata* flies from *E*. *amylovora* contaminated mature apples to healthy ones. Data are from two independent experiments with two replicates each. The females transmitted *E*. *amylovora* cells in significantly higher numbers (p<0.05) to the distal parts of the fruit, either the peduncle area or the calyx region.

Pathogen transmission to the different fruit parts and the possible relationship between transmission and the sex of the medfly were further studied. Regardless of the medfly sex, the majority of the *E*. *amylovora* external population transmitted was found in the peel surrounding the calyx and in the area of the greatest diameter of the fruit (Fig 2A). In contrast, the internal population was mainly found in the peduncle area (Fig 2A). The males transmitted *E*. *amylovora* cells in significantly higher numbers (p<0.05) to the peel than to the flesh of the fruit, but no significant differences (p>0.05) between these two parts were found for the females (S2 Fig). Moreover, significant differences (p<0.05) between the sexes were also observed inside the distal parts of the fruit (Fig 3), where the transmission was mainly due to females, either in the peduncle area or in the calyx region (Fig 2A). Data on the incidence of detection are not provided in Fig 2 because *E*. *amylovora* was detected in all the samples with positive results and in none with negative ones.

#### (ii) Transmission to pear shoots and plants

In transmission assays of *E*. *amylovora* from contaminated apple fruits to detached pear shoots, irregular necrotic lesions of various sizes (0.5–4 cm) were observed on several leaves of each shoot after 5–7 days of contact with the medflies (Fig 2B and 2C). The severity of the lesions was apparently independent of whether the leaves had been injured or not. Some egg clusters were occasionally noticed as being embedded in the leaf tissues and surrounded by a necrotic margin (Fig 2D). The presence of *E*. *amylovora* in the leaf necrotic areas and in the transmitting medflies was confirmed by cultural and PCR techniques, with higher populations of *E*. *amylovora* recovered from the pear leaf lesions (10^5^–10^7^ CFU/necrotic lesion) than from the transmitting medflies (less than 10 CFU/fly). The results were negative for egg samples.

In assays of transmission to potted pear plants, slight necrotic symptoms were observed on several leaves of each plant, showing between 1–4 dark necrotic spots (approximately 0.2 cm diameter) per leaf after 10–14 days in contact with contaminated medflies (Fig 2E). Some leaves were punctured by female medflies to lay their eggs. Spots showed a random distribution, being observed in either young or adult, injured or intact leaves, and on the upper or the underside of leaf surfaces, and *E*. *amylovora* was isolated from these spots. No *E*. *amylovora*-like colonies were recovered from the medflies after 14 days of contact with the bacterium, which is in accordance with the survival results. However, the PCR results for *E*. *amylovora* were positive for both pear leaves and transmitting medflies from all of the samples assayed, but not for the eggs deposited by the flies.

**Fig 4:**
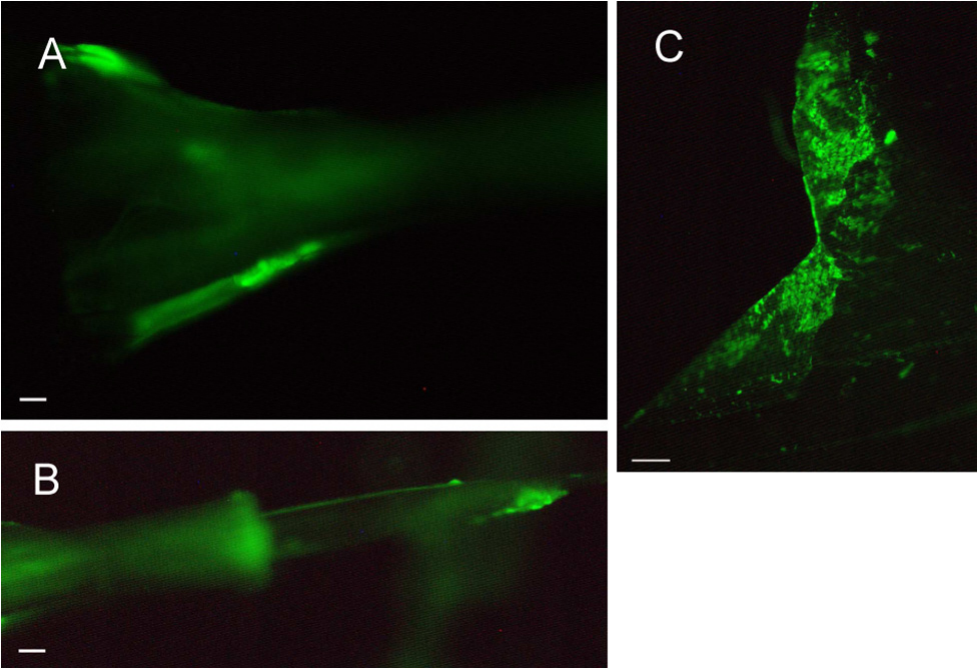
Location of *E*. *amylovora* cells on *C*. *capitata* female. CFBP1430 strain cells tagged with GFP protein monitored on a female of *C*. *capitata* after the transmission period (5 days) on the 7^**th**^ abdominal segment (A), the 9^**th**^ abdominal segment (the end of the ovipositor) (B), and the wings (C). White bar represents 10 μm in A-B and 100 μm in C.

Green fluorescent *E*. *amylovora* cells, regardless of the assayed strain, were mostly found as cellular aggregates on the ovipositors of medfly females (Fig 4A and 4B) and on the distal parts of the wings of both sexes (Fig 4C) after transmission challenge.

### Location of *E*. *amylovora* into medflies

**Fig 5:**
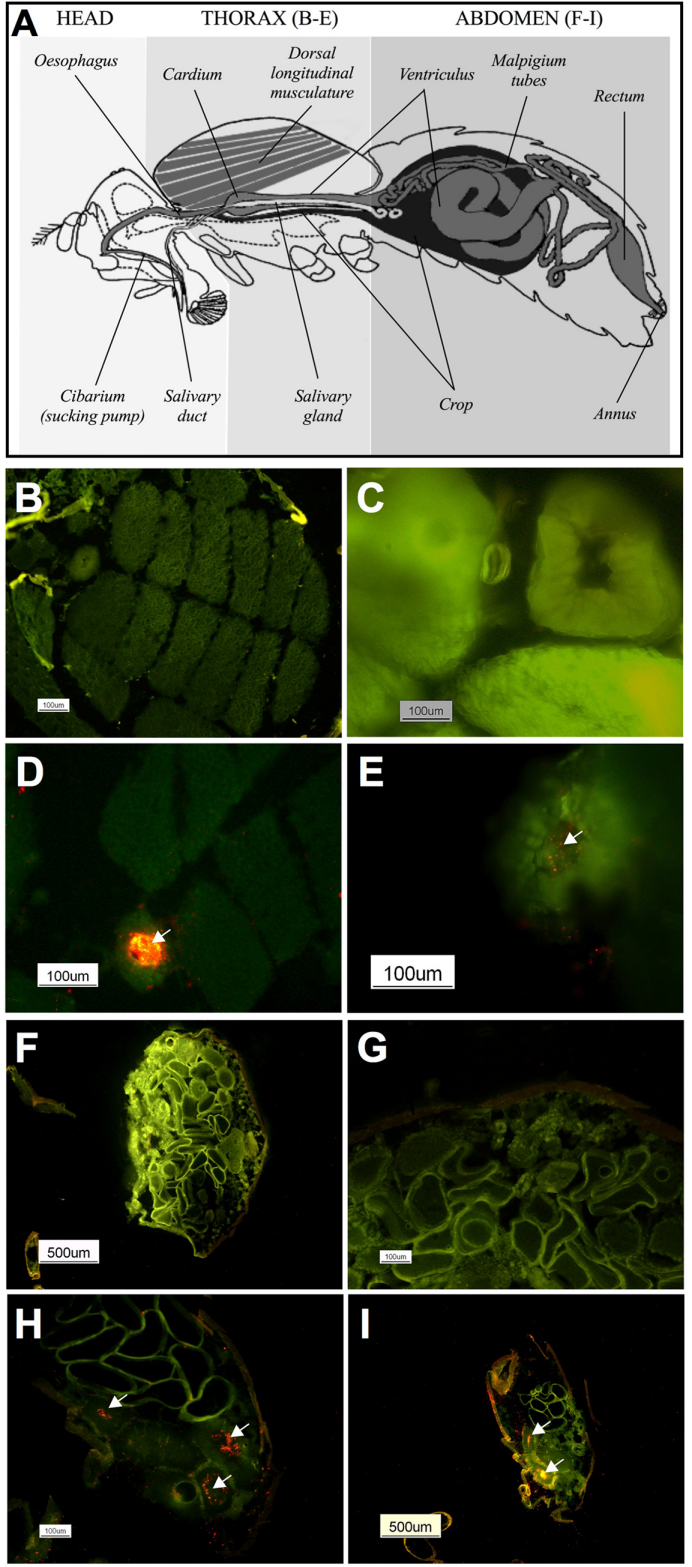
Location of *E*. *amylovora* cells into the *C*. *capitata* body. The dorsal longitudinal thoracic flight muscles and the digestive system in an adult fly (A) and cross sections of thorax (B-E) and abdomen (F-I) of control (B, C, F, G) or challenged (D, E, H, I) female medflies fed with red fluorescent *E*. *amylovora* NCPPB 2080 cells for 24h (D, H) and 4 days (E, I) after the acquisition period.

Red fluorescent cells were observed in the mouth of medflies challenged with red-fluorescent tagged bacteria, not only on the surface but also in the inner parts; they were not found in medflies fed with wild type bacterial strains (data not shown). Fig 5A shows a diagram of the thorax and abdomen of medflies. In contrast to the observations in the negative control medflies (Fig 5B and 5C), red-fluorescent bacteria were observed in thorax and abdomen sections of challenged insects, inside the digestive tract (Fig 5D and 5E), both in the lumen (Fig 5D) and/or coating the internal wall of the tube (Fig 5E). Moreover, red-fluorescent bacteria were also located in the crop of challenged medflies (data not shown). Fig 5F and 5G show control flies with no *E*. *amylovora* cells in the abdomen; in this structure, in general, the amount of red-fluorescent bacteria in challenged flies was higher than in thorax (Fig 5H and 5I), and mostly the bacterial cells appeared as aggregates and extended by wide areas along the abdomen. In all cases, red bacteria were observed in these structures, both in thorax and abdomen, until 8 days after the acquisition period. At 15 days, all analyzed samples were negative for the presence of red-fluorescent bacteria, and the enrichment did not allow the detection of the target pathogen. Interestingly, red-fluorescent cells were not observed inside ovipositor structure.

## Discussion

The spread of plant pathogens to new hosts by insect vectors can cause increased damage and economic losses [1]. Although there are many insects associated with *E*. *amylovora* dissemination [10], their exact role and the bacterial location and survival in/on the insects has not been studied in depth and only by isolation techniques. More specifically, the role of non-pollinating insects is poorly understood [4]. Consequently, there is a lack of knowledge on the role of these insects as potential fire blight vectors, in spite they are recognized as a contributing factor in the epidemiology of this disease [3]. Past research on flies showed that the vinegar fly (*Drosophila melanogaster*) could be involved in the spread of *E*. *amylovora* [7]. Recently, the transport of *E*. *amylovora* cells on greenbottle flies to wounded young fruit and pear shoot was demonstrated under experimental conditions, but no information on the bacterial location in/on the flies was provided [3]. Despite *C*. *capitata*’s relevance as worldwide fruit pest of high economic importance [13, 14, 15], its potential role on *E*. *amylovora* transmission has not been studied before.

Pathogen transmission by contaminated insects depends not only on the pathogen’s dose carried by them but also on the pathogen’s survival period in/on the insect [8]. In this study, *E*. *amylovora* showed a notable ability to persist in a culturable state in/on *C*. *capitata* for up to 14 days. On honeybees [7, 8, 41, 42, 43] *E*. *amylovora* was reported to survive only up to 6 days [7]. Nevertheless, the persistence of *E*. *amylovora* in/on insects is likely to have been underestimated due to two reasons: survival has been assessed only by culturability on solid media and no studies have investigated its survival inside insects. In our experiments, after 14 days in contact with the fly, *E*. *amylovora*-like colonies were not isolated. This could indicate either bacterial cell death or loss of culturability. It is remarkable that the lack of *E*. *amylovora* colonies ruled out the possibility that repetitive contacts of the flies with the sucrose solution, used for feeding, led to its contamination with the bacterium. Consequently, this excludes the feed as a source of re-introduction or redistribution of the pathogen on flies over time. Moreover, the negative result of the enrichment in CCT from the challenged cages (food, water, walls) also indicated that insects could not acquire the target bacteria continuously, supporting the significance of the data on *E*. *amylovora* acquisition by *C*. *capitata*.

To elucidate whether non-culturable *E*. *amylovora* cells were dead or alive after 14 days of bacterium-medfly contact, a recovery method previously described [24, 33, 37] was challenged to demonstrate regained culturability. Although the KB liquid medium had provided excellent results for recovering non-culturable *E*. *amylovora* cells induced by certain stress conditions, neither it nor CCT broth were successful here. The passage through susceptible host plant material [37], however, resulted an appropriate method. The inoculation of medfly extracts containing non-culturable *E*. *amylovora* cells into susceptible pear plants caused necrotic lesions from which the pathogen was subsequently re-isolated. These results, which confirm *E*. *amylovora*’s survival ability in non-host environments [32, 33, 37, 44, 45, 46, 47, 48, 49, 50], also demonstrate that stressed or injured bacterial cells [28, 37, 51] can remain viable on the medfly, for at least 28 days, and can be potentially pathogenic. This is the longest survival period reported for *E*. *amylovora* on any insect [7, 41]. Consequently, this integrated protocol, based on isolation, molecular and bioassay approaches, not applied before to insects, is highly useful to recover bacterial cells and should be further applied to honeybees and other insects.

Furthermore, transmission experiments revealed that *C*. *capitata* was able to efficiently acquire *E*. *amylovora* from contaminated mature apples (inoculated with approximately 10^5^–10^6^ CFU per fruit) and transmit it to healthy ones, consistently in all the assays performed. The assayed *E*. *amylovora* strains (CFBP1430 and NCPPB2080) had been described as highly and moderately aggressive, respectively [52], and one viable cell of *E*. *amylovora* is enough to develop an infection under highly favorable conditions [52]. The fact that the bacterium-recipient apples remained asymptomatic was most likely due to *E*. *amylovora*’s inability to multiply on ripe fruit [24, 36, 53]. Nevertheless, the pathogen was recovered from both the apple’s peel and flesh, suggesting a pathogen-insect relationship probably involving insect reproductive and digestive processes [20, 54, 55]. This hypothesis was confirmed by microscopic examinations where *E*. *amylovora* red-fluorescent cells were observed in the digestive tract along the thorax and abdomen of the medflies 8 days after the acquisition period. This finding is unknown up to now and very relevant for fire blight epidemiology, as there was no previous evidence of *E*. *amylovora* internalization by insects [2].

Both medfly sexes contributed equally to pathogen transmission in the apple peel. The presence of fluorescent (GFP-marked) cells on the wings of flies of both sexes is most likely related to the fly’s position during feeding, defecating, or laying eggs (females), enabling contact between the distal parts of the wings with the fruit or leaf. The transmission to the interior of the fruit was mainly due to female insects. The observation of GFP cells on the external surface of ovipositors of transmitting medflies coincided with the detection of *E*. *amylovora* cells inside the apple flesh. These were probably introduced by laying of eggs and/or fecal contamination [54, 56]. The female medfly may lay approximately 20 eggs per day, in egg-clutches of 1 to 10 eggs, by puncturing the fruit up to 0.5 cm deep with its ovipositor [57]. Indeed, some tunnels containing eggs were found in the flesh of several bacterium-recipient apples. However, *E*. *amylovora* was never detected in eggs. These results agree with the findings of other studies where vertical transmission of plant pathogens (mother to egg or sperm to egg) is unusual in insect vectors [58, 59]. The contamination was most likely external, on the surface of the ovipositor. In fact, red-fluorescent *E*. *amylovora* cells were not found inside the ovipositor structure in any sample. Moreover, it is known that *C*. *capitata* usually produces two ceratoxins to prevent the presence of bacteria in the ovaries [56].

Assays of transmission to susceptible plant materials other than fruit, including detached young shoots and whole pear plants, revealed that the transmitted pathogen could effectively reach host tissues, causing leave necrosis of variable extent. Although egg laying on leaves or penetration in leaf tissue is not the normal behavior of medflies, when fruit is not available flies can lay eggs on other plant material [14].

One of the goals of this work was to elucidate the ability of medflies to transmit fire blight pathogen to shoots or other organs, since it is less understood than the blossom blight transmission [4]. Under our experimental conditions, *E*. *amylovora* can survive in/on *C*. *capitata* and be efficiently transmitted from mature apples to different healthy plant materials, without requiring artificial wounds in plant tissue. This transmission could go unnoticed because of the absence of fire blight symptoms [24] or the presence of atypical disease symptoms in leaves. Medfly should not be neglected as a key ecological player, as it is likely to have repeated encounters with *E*. *amylovora* in different parts of the host plants, so flies travelling from plant to plant could act as vectors, as shown for other insects [60]. There are many species of flies that may be present in apple or pear orchards, some considered non-pests and others causing damage, such as *Rhagoletis pomonella* (Walsh) [17] or *Drosophila suzukii* (Diptera: Drosophilidae) [61], but *C*. *capitata* remains the pest most widely distributed in these orchards throughout the year [15]. Although the role of other fly species in fire blight epidemiology is still unknown, our results with *C*. *capitata* suggest other fly species could also be efficient vehicles for *E*. *amylovora* transmission.

Overall, the presence of *C*. *capitata* in fruit-growing areas with fire blight should not be disregarded as a potential threat for dissemination of *E*. *amylovora*. The exceptional ability of *E*. *amylovora* to survive in *C*. *capitata*, both in the medfly surface and inside its digestive tract, the efficiency of this insect in acquiring and transmitting this bacterium, which remains pathogenic, and the relatively high cell numbers of the plant pathogen carried by each medfly in our experimental system demonstrate its potential as a fire blight vector. A clear understanding of medfly role in dissemination of *E*. *amylovora* and on the epidemiology of fire blight, with field studies in orchards with infected hosts, will contribute to design more optimized control strategies.

## Supporting Information

S1 Fig Boxplot of CFU mL^-1^ of *E*. *amylovora* from *Ceratitis capitata* flies after acquisition experiments. *E*. *amylovora*-like colonies, confirmed by PCR, were recovered after 7 and 14 days of contact of medflies with *E*. *amylovora* contaminated apples. Data are from two independent experiments with two replicates each. There were no significant differences among the four assayed strains (p>0.05).
(TIF)

S2 Fig Boxplot of CFU mL^-1^ of *E*. *amylovora* from surface or flesh of mature apples after transmission by *C*. *capitata* flies. Colonies of CFBP1430 strain counted from apple surface or flesh after transmission by male or female flies from *E*. *amylovora* contaminated mature apples to healthy ones. Data are from two independent experiments with two replicates each. The males transmitted *E*. *amylovora* cells in significantly higher numbers (p<0.05) to the peel than to the flesh of the fruit.
(TIF)

## References

[1] Agrios GN (2009) Transmission of plant diseases by insects. Available: http://entomology.ifas.ufl.edu/capinera/eny5236/pest1/content/03/3_plant_diseases.pdf

[2] Nadarasah G, Stavrinides J (2011) Insects as alternative hosts for phytopathogenic bacteria. FEMS Microbiol Rev 35: 555–575. 10.1111/j.1574-6976.2011.00264.x 21251027

[3] Thomson SV (2000) Epidemiology of fire blight In: Vanneste JL, editor. Fire blight: The disease and its causative agent, *Erwinia amylovora*. Wallingford, United Kingdom: CABI Publishing pp. 9–36.

[4] van der Zwet T, Beer SV (1995) Fire blight—Its nature, prevention and control A practical guide to integrated disease management. Agriculture Information Bulletin N° 631. Washington D.C., USA: USDA.

[5] Billing E (2011) Fire blight. Why do views on host invasion by *Erwinia amylovora* differ? Plant Pathol. 60: 178–189.

[6] Vanneste JL, Eden-Green S (2000) Migration of *Erwinia amylovora* in host plant tissue Fire blight: the disease and its causative agent, *Erwinia amylovora* (Vanneste JL, ed.), pp. 73–83. Wallingford, UK: CAB International.

[7] Ark PA, Thomas HE (1936) Persistence of *Erwinia amylovora* in certain insects. Phytopathology 26: 375–381.

[8] Hildebrand M, Dickler E, Geider K (2000) Ocurrence of *Erwinia amylovora* on insects in a fire blight orchard. J Phytopathol 148: 251–256.

[9] Schroth MN, Thomson SV, Hildebrand DC (1974) Epidemiology and control of fire blight. Annu Rev Phytopathol 12: 389–412.

[10] van der Zwet T, Keil HL (1979) Fire blight: A bacterial disease of roseaceous plants. United States Department Agriculture Handbook 510, Washington DC, USA.

[11] van der Zwet T, Orolaza-Halbrendt N, Zeller W (2012) Fire blight: history, biology, and management APS Press/American Phytopathological Society.

[12] Cayol JP, Causse R (1993) Mediterranean fruit fly *Ceratitis capitata* Wiedemann (Dipt., *Trypetidae*) back in Southern France. J Appl Entomol 116: 94–100.

[13] Aluja M, Liedo P (1993) Fruit flies: biology and management New York: Springer,. 492 pp.

[14] Aluja M, Mangan RL (2008) Fruit fly (Diptera: Tephritidae) host status determination: critical conceptual, methodological, and regulatory considerations. Annu Rev Entomol 53: 473–502. 17877455 10.1146/annurev.ento.53.103106.093350

[15] Aluja M, Norrbom AL (2000) Fruit flies (Tephritidae): phylogeny and evolution of behavior Boca Raton, Florida: CRC. 944 pp.

[16] EPPO, European and Mediterranean Plant Protection Organization (1981) Data sheets on quarantine organisms No. 105, *Ceratitis capitata* . OEPP/EPPO Bull. 11: 1.

[17] White IM, Elson-Harris MM (1994) Fruit flies of economic significance: their identification and bionomics Oxon, UK: CAB International. 601 p.

[18] Israely N, Ziv Y, Calun R (2005) Metapopulation spatial-temporal distribution patterns of Mediterranean fruit fly (Diptera: Tephritidae) in a patchy environment. Ann Entomol Soc Am 98 (3): 302–308.

[19] Meats A, Smallridge CJ (2007) Short- and long-range dispersal of medfly, *Ceratitis capitata* (Dipt., Tephritidae), and its invasive potential. J Appl Entomol 131(8): 518–523.

[20] Cayol JP, Causse R, Louis C, Barthes J (1994) Medfly *Ceratitis capitata* Wiedemann (Dipt., *Trypetidae*) as a rot vector in laboratory conditions. J Appl Entomol 117: 338–343.

[21] Janisiewicz WJ, Conway WS, Brown MW, Sapers GM, Fratamico P, Buchanan RL (1999) Fate of *Escherichia coli* O157:H7 on fresh cut apple tissue and its potential for transmission by fruit flies. Appl Environ Microbiol 65: 1–5. 9872751 10.1128/aem.65.1.1-5.1999 PMC90974

[22] Sela S, Nestel D, Pinto R, Nemny-Lavy E, Bar-Joseph M (2005) Mediterranean fruit fly as a potential vector of bacterial pathogens. Appl Environ Microbiol 71: 4052–4056. 16000820 10.1128/AEM.71.7.4052-4056.2005 PMC1169043

[23] Juan-Blasco M, Sabater-Muñoz B, Argiles R, Jacas JA, Castañera P, Urbaneja A (2013) Molecular tools for sterile sperm detection to monitor *Ceratitis capitata* populations under SIT programmes. Pest Man. Sci. 69: 857–864. 10.1002/ps.3448 23355333

[24] Ordax M, Biosca EG, Wimalajeewa SC, López MM, Marco-Noales E (2009) Survival of *Erwinia amylovora* in mature apple fruit calyces through the viable but non-culturable (VBNC) state. J Appl Microbiol 107: 106–116. 10.1111/j.1365-2672.2009.04187.x 19298508

[25] Taylor RK, Hale CN, Gunson FA, Marshall JW (2003) Survival of the fire blight pathogen, *Erwinia amylovora*, in calyxes of apple fruit discarded in an orchard. Crop Prot 22: 603–608.

[26] van der Zwet T, Thomson SV, Covey RP, Bonn WG (1990) Populations of *Erwinia amylovora* on external and internal apple fruit tissues. Plant Dis 74: 711–716.

[27] Ishimaru C, Klos EJ (1984) New medium for detecting *Erwinia amylovora* and its use in epidemiological studies. Phytopathology 74: 1342–1345.

[28] Ordax M, Biosca EG, López MM, Marco-Noales E (2012) Improved recovery of *Erwinia amylovora* stressed cells from pome fruit on RESC, a simple, rapid and differential medium. Trees 26: 83–93.

[29] King EO, Ward M, Raney DE (1954) Two simple media for the demonstration of pyocyanin and fluorescein. J Lab Clin Med 44: 401–407. 13184240

[30] Lelliot RA (1967) The diagnosis of fire blight (*Erwinia amylovora*) and some diseases caused by *Pseudomonas syringae* . Bull OEPP 45: 27–34.

[31] San Andrés V, Urbaneja A, Sabater-Muñoz B, Castañera P (2007) A novel molecular approach to assess mating success of sterile *Ceratitis capitata* (Diptera: Tephritidae) males in sterile insect technique programs. J Econ Entomol 100: 1444–1449. 17849900 10.1603/0022-0493(2007)100[1444:anmata]2.0.co;2

[32] Santander RD, Oliver JD, Biosca EG (2014). Cellular, physiological and molecular adaptive responses of *Erwinia amylovora* to starvation. FEMS Microbiol Ecol 88: 258–271. 10.1111/1574-6941.12290 24476337

[33] Ordax M, Marco-Noales E, López MM, Biosca EG (2006) Survival strategy of *Erwinia amylovora* against copper: induction of the viable-but-nonculturable state. Appl Environ Microbiol 72: 3482–3488. 16672494 10.1128/AEM.72.5.3482-3488.2006 PMC1472350

[34] Taylor RK, Guilford PJ, Clark RG, Hale CN, Forster RLS (2001) Detection of *Erwinia amylovora* in plant material using novel polymerase chain reaction (PCR) primers. N Z J Crop Hortic Sci 29: 35–43.

[35] Juan-Blasco M, San Andrés V, Martínez-Utrillas MA, Argilés R, Pla I, Urbaneja A, et al (2013) Alternatives to ginger root oil aromatherapy for improved mating performance of sterile *Ceratitis capitata* (Diptera: Tephritidae) males. J Appl Entomol 137(s1): 244–251.

[36] EPPO, European and Mediterranean Plant Protection Organization (2013) Diagnostic protocols for regulated pests. Phytosanitary mesures PM7/20 *Erwinia amylovora* . Bull OEPP/EPPO Bull 43: 21–45.

[37] Santander RD, Catalá-Senent J, Marco-Noales E, Biosca EG (2012) *In planta* recovery of *Erwinia amylovora* viable but non-culturable cells. Trees 26: 75–82.

[38] Gorris MT, Cambra M, Lecomte P, Llop P, Chartier R, Paulin JP, et al (1996) A sensitive and specific detection of *Erwinia amylovora* based on ELISA-DASI enrichment method with monoclonal antibodies. Acta Hort 411: 41–46.

[39] Llop P, Caruso P, Cubero J, Morente C, López MM (1999) A simple extraction procedure for efficient routine detection of pathogenic bacteria in plant material by polymerase chain reaction. J Microbiol Methods 37: 23–31. 10395461 10.1016/s0167-7012(99)00033-0

[40] Suckling DM, Woods D, Mitchell VJ, Twidle A, Lace I, Jan EB, et al (2011) Mobile mating distruption of light-brown apple moths using pheromone-treated sterile Mediterranean fruit flies. Pest Man. Sci. 67(8): 1004–1014. 10.1002/ps.2150 21480460

[41] Alexandrova M, Cimini B, Bazzi C, Carpana E, Massi S, Sabatini AG (2002) The role of honeybees in spreading *Erwinia amylovora* . Acta Hort 590: 55–60.

[42] Miller TD., Schroth MN (1972) Monitoring the epiphytic population of *Erwinia amylovora* on pear with a selective medium. Phytopathology 62: 1175–1182.

[43] Tsukamoto T, Uematsu H, Mizuno A, Kimura S (2005) Transmission of *Erwinia amylovora* from blighted mature apple fruit to host plants via flies. Res Bull Plant Prot Serv Jpn 41: 65–70.

[44] Biosca EG, Santander RD, Ordax M, Marco-Noales E, López MM (2008) *Erwinia amylovora* survives in natural water. Acta Hort 793: 83–87.

[45] Biosca EG, Santander RD, Ordax M, Marco-Noales E, Águila B, Flores A, López MM (2009) Survival of *Erwinia amylovora* in rain water at low temperatures, p 88–91. In: Mendez-Vilas A, editor. Current Research Topics in Applied Microbiology and Microbial Biotechnology. Singapore: World Scientific Publishing Co. Pte. Ltd.

[46] Ceroni P, Minardi P, Babini V, Traversa F, Mazzucchi U (2004) Survival of *Erwinia amylovora* on pears and on fruit containers in cold storage and outdoors. Bull OEPP/EPPO Bull 34: 109–115.

[47] Hildebrand M, Tebbe CC, Geider K (2001) Survival studies with the fire blight pathogen *Erwinia amylovora* in soil and in a soil-inhabiting insect. J Phytopathol 149: 635–639.

[48] Ordax M, Marco-Noales E, López MM, Biosca EG (2010) Exopolysaccharides favor the survival of *Erwinia amylovora* under copper stress through different strategies. Res Microbiol 161: 549–555. 10.1016/j.resmic.2010.05.003 20546893

[49] Santander RD, Monte-Serrano M, Rodríguez-Herva J, López-Solanilla E, Rodríguez-Palenzuela P, Biosca EG (2014). Exploring new roles for the *rpoS* gene in the survival and virulence of the fire blight pathogen *Erwinia amylovora* . FEMS Microbiol Ecol 90: 895–907. 10.1111/1574-6941.12444 25331301

[50] Southey RFW, Harper GJ (1971) The survival of *Erwinia amylovora* in airborne particles: tests in the laboratory and in the open air. J Appl Bacteriol 34: 547–556.

[51] Liao C, Shollenberger LM (2004) Enumeration, resuscitation, and infectivity of the sublethally injured *Erwinia* cells induced by mild acid treatment. Phytopathology 94: 76–81. 10.1094/PHYTO.2004.94.1.76 18943822

[52] Cabrefiga J, Montesinos E (2005) Analysis of aggressiveness of *Erwinia amylovora* using disease-dose and time relationships. Phytopathology 95: 1430–1437. 10.1094/PHYTO-95-1430 18943554

[53] Dueck J (1974) Survival of *Erwinia amylovora* in association with mature apple fruit. Can J Plant Sci 54: 349–351.

[54] Behar A, Ben-Yosef M, Lauzon CR, Yuval B, Jurkevich E (2009) Structure and function of the bacterial community associated with the Mediterranean fruit fly In: Bourtzis K, Miller T, editors. Insect Symbiosis, vol. 3 Boca Raton, Florida: CRC Press pp. 251–271.

[55] Coronado-González PA, Vijaysegaran S, Robinson AS (2008) Functional morphology of the mouthparts of the adult Mediterranean fruit fly, *Ceratitis capitata* . J Insect Sci 8: 73.

[56] Behar A, Jurkevitch E, Yuval B (2008) Bringing back the fruit into fruit fly-bacteria interactions. Mol Ecol 17: 1375–1386. 10.1111/j.1365-294X.2008.03674.x 18302695

[57] McDonald PT, McInnis DO (1985) *Ceratitis capitata*: effect of host fruit size on the number of eggs per clutch. Entomol Exp Appl 37 (3): 207–211.

[58] Mann RS., Pelz-Stelinski K, Hermann SL, Tiwari S, Stelinski LL (2011) Sexual transmission of a plant pathogenic bacterium, *Candidatus* Liberibacter asiaticus, between conspecific insect vectors during mating. PLOS One 6 (12): e29197 10.1371/journal.pone.0029197 22216209 PMC3244449

[59] Purcell AH (1982) Insect vector relationships with prokaryotic plant pathogens. Annu Rev Phytopathol 20: 97–417.

[60] Emmet BJ, Baker LAE (1971) Insect transmission of fireblight. Plant Pathol 20: 41–45.

[61] EPPO, European and Mediterranean Plant Protection Organization (2010) Data sheets on quarantine organisms No. 12–2010, *Drosophila suzukii* http://www.eppo.org/QUARANTINE/Alert_List/insects/Drosophila_suzukii_factsheet_12-2010.pdf

