# Supplementary figures and images for: Medfly Ceratitis capitata as Potential Vector for Fire Blight Pathogen Erwinia amylovora: Survival and Transmission
Source: PLoS One. 2015 May 15;10(5):e0127560. doi: 10.1371/journal.pone.0127560 (PMC4433354; doi:10.1371/journal.pone.0127560)

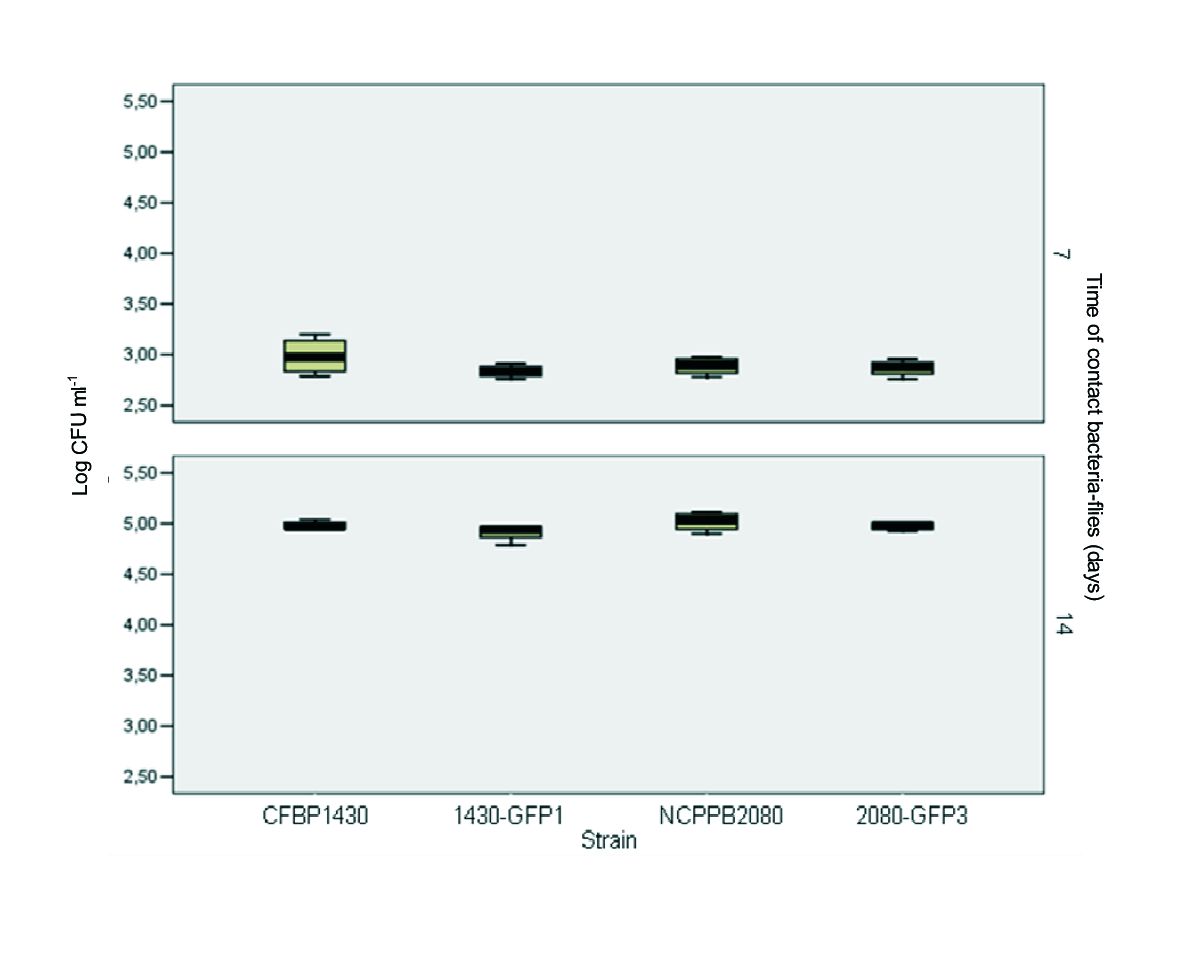

Supplement: S1 Fig — E. amylovora-like colonies, confirmed by PCR, were recovered after 7 and 14 days of contact of medflies with E. amylovora contaminated apples. Data are from two independent experiments with two replicates each. There were no significant differences among the four assayed strains (p>0.05). (TIF) [file pone.0127560.s001.tif]

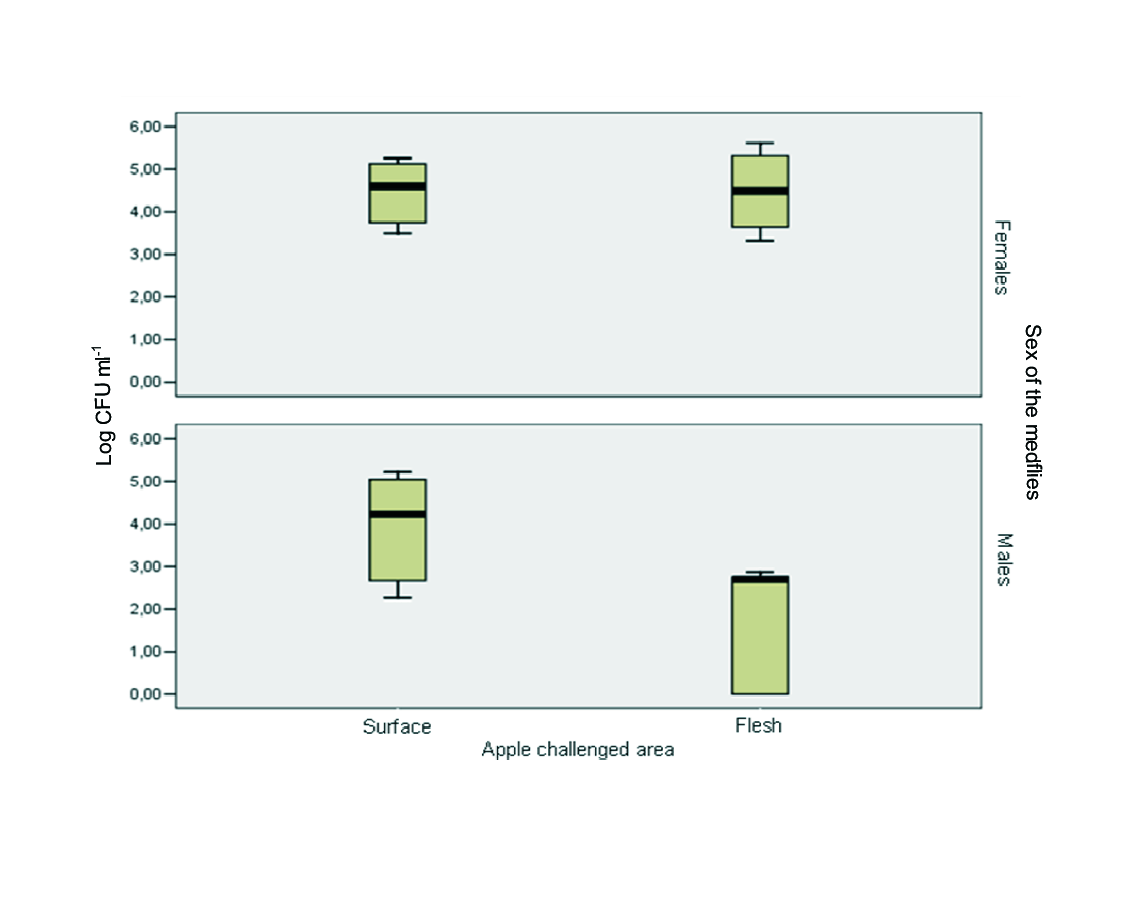

Supplement: S2 Fig — Colonies of CFBP1430 strain counted from apple surface or flesh after transmission by male or female flies from E. amylovora contaminated mature apples to healthy ones. Data are from two independent experiments with two replicates each. The males transmitted E. amylovora cells in significantly higher numbers (p<0.05) to the peel than to the flesh of the fruit. (TIF) [file pone.0127560.s002.tif]
